# The Role of Microbiota in Infant Health: From Early Life to Adulthood

**DOI:** 10.3389/fimmu.2021.708472

**Published:** 2021-10-07

**Authors:** Yao Yao, Xiaoyu Cai, Yiqing Ye, Fengmei Wang, Fengying Chen, Caihong Zheng

**Affiliations:** ^1^ Department of Pharmacy, Women’s Hospital School of Medicine Zhejiang University, Hangzhou, China; ^2^ Department of Clinical Pharmacology, Key Laboratory of Clinical Cancer Pharmacology and Toxicology Research of Zhejiang Province, Affiliated Hangzhou First People’s Hospital, Cancer Center, Zhejiang University School of Medicine, Hangzhou, China

**Keywords:** microbiome, maternal, offspring, newborn, pregnancy, long-term health, brain development, immune system development

## Abstract

From early life to adulthood, the microbiota play a crucial role in the health of the infant. The microbiota in early life are not only a key regulator of infant health but also associated with long-term health. Pregnancy to early life is the golden time for the establishment of the infant microbiota, which is affected by both environmental and genetic factors. Recently, there is an explosion of the studies on the role of microbiota in human diseases, but the application to disease or health is relatively limited because many aspects of human microbiota remain controversial, especially about the infant microbiota. Therefore, a critical and conclusive review is necessary to understand fully the relationship between the microbiota and the health of infant. In this article, we introduce in detail the role of microbiota in the infant from pregnancy to early life to long-term health. The main contents of this article include the relationship between the maternal microbiota and adverse pregnancy outcomes, the establishment of the neonatal microbiota during perinatal period and early life, the composition of the infant gut microbiota, the prediction of the microbiota for long-term health, and the future study directions of microbiota.

## 1 Introduction

The emergence of the concept of the microbiome and the development of molecular technology, especially 16S rRNA, have greatly increased the understanding of the microbiota in maternal-fetal interface and early life (**Table 1** for glossary of terms). This makes it possible to characterize the microbiota of pregnant women and their offspring without culturing. During pregnancy, the maternal microbiota affects the development of the fetus, especially the brain development, such as the uterine microbiota, the vagina microbiota, the gastrointestinal microbiota, the placental microbiota (controversial), and the oral microbiota. Notably, the disorder of the maternal microbiota can lead to adverse pregnancy outcomes (APOs), which seriously threaten the health of the offspring. After birth, the infant microbiota affected by both environmental and genetic factors are quickly established to ensure healthy growth. In this article, we describe the role of the microbiota (including the maternal microbiota during pregnancy and the infant microbiota during early life) in the health of offspring from pregnancy to long-term health. These provide a theoretical basis for improving infant health by adjusting the microbiota in pregnancy and early life and promote the understanding of maternal and fetal health.

**Table 1:** Glossary of terms.

Terms	Description
Microbiota	Microbial community in a particular environment. May be used interchangeably with “microbiome.”
Microbiome	Combined genetic material of microorganisms in a particular environment. May be used interchangeably with “microbiota.”
Microbe	A microorganism, especially a bacterium causing disease or fermentation.
Diversity	The range of different types of organisms and their relative abundance in a particular environment.
16S rRNA	16S ribosomal RNA is a subunit of the ribosomal RNA containing specific signature sequences useful for bacterial identification.
Abundance	Relative level of a microbe

The search strategy of this article: We search literature and collect data through Web of Science and Pubmed. First, we use different keyword combinations to search, including infant or newborn or offspring, microbiota* or microbiome* or microbe*, maternal, pregnancy or pregnant or gestation*, etc. Secondly, we read the retrieved articles and eliminate irrelevant articles. Then, we read the rest of the literature carefully and developed a manuscript outline. Finally, we extract the target content from the literature through summary and induction.

## 2 The Maternal Microbiota and Offspring During Pregnancy

### 2.1 The Maternal Microbiota During Pregnancy

**Figure 1:**
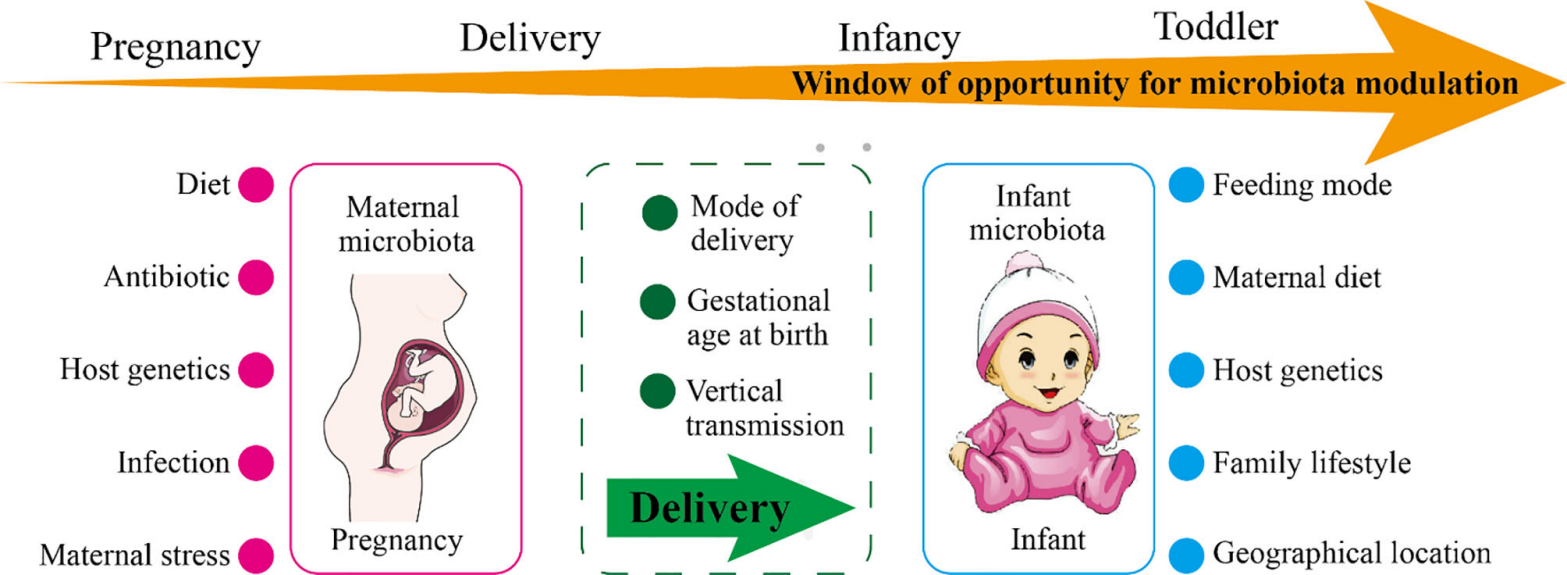
The factors affecting maternal or infant microbiota and the windows of opportunity for microbiota modulation. As shown in the figure, the maternal microbiota and the infant microbiota are affected by environmental and genetic factors. During pregnancy, the main influencing factors of the maternal microbiota are diet, use of antibiotics, infection, stress, and host genetics. During delivery, the maternal microbiota will be transmitted vertically to the newborn, and the delivery mode and the gestational age of the newborn at birth affect the colonization of the newborn’s microbiota. The main influencing factors of infant microbiota are feeding mode, maternal diet, family lifestyle, geographic location, and host genetics. The window of opportunity for offspring microbiota modulation are pregnancy, delivery, infancy, and toddler.

Previously, the culprit of intrauterine infections in the fetus was considered to be the microbes that rise from the vagina, such as bacteria, viruses, and fungi. However, with the development of science and technology, the oral and gut microbiota have been found to be also associated with the health of the fetus because the microbiota in these parts can spread through the blood. During pregnancy, the mother’s gut, oral cavity, and vagina microbiota all undergo changes. These changes are related to a variety of factors, including diet, antibiotic use, infections, stress, and host genes (**Figure 1**) (1–5). The study of Romero et al. revealed that the stability of the vaginal microbiota of healthy pregnant women is higher than that of healthy nonpregnant women and *Lactobacillus* species were the main component of the vaginal microbiota of healthy pregnant women (6). Traditionally, the uterus is considered to be sterile, but this concept was broken until recent years. The endometrium has its own microbiota, and the biomass of these microbiota is very low (7). Regrettably, the current understanding of the endometrium microbiota is limited, and its role in fetal development and pregnancy outcome still needs to be fully elucidated.

Emerging evidence indicated that the richness and homogeneity of the gut microbiota of pregnant women are not significantly different from those of normal women, but the distribution and composition are changed obviously (8). In addition, pregnant women with pregnancy complications have a reduced diversity of their gut microbiota, which is detrimental to the health of both the mother and the fetus. For example, gut microbial remodeling during pregnancy in rats with chronic hypertension is impaired (9). The abundance of short-chain fatty acid-producing *Coprococcus* in the gut microbiota of pregnant women with preeclampsia is decreased (10). In 2014, Aagaard et al. found that the human placental microbiota are mainly composed of nonpathogenic symbiotic microbiota, including *Fusobacteria* phyla, *Firmicutes*, *Bacteroidetes*, *Tenericutes*, and *Proteobacteria* (11). The human placental microbiota are similar to the oral microbiota and limited diversity compared with the gut microbiota (11, 12). However, on the contrary, another study showed that the human placenta does not have microbiota but there may be potential pathogens (13). Also, the study concluded there is no correlation between bacterial infection of the placenta and common adverse pregnancy outcomes (APOs) (13).

The imbalance of the maternal microbiota leads to a variety of complications during pregnancy, such as obesity, hypertension, and preeclampsia. Here, we mainly introduce the impact of the maternal microbiota on the fetus, so we will not elaborate on the complications of pregnancy. The differential change of the maternal microbiota during pregnancy is an important cause of APOs. In the following paragraphs, we introduce the factors that affect the maternal microbiota and the impact of the maternal microbiota on APOs.

### 2.2 The Factors Affecting Maternal Microbiota During Pregnancy

At present, the factors that have been clearly proven to affect the microbiota of the vagina and other parts of pregnant women are diet, antibiotic use, infection, and maternal stress. Factors that need to be further studied are immune status, age, and genetic background. In addition to these factors, complications during pregnancy are also important influencing factors. For example, the changes in the composition of the gut microbiota, oral microbiota, and vaginal microbiota in patients with gestational diabetes are obvious (14). Pregnancy is one of the important windows for regulating the microbiota, and the influencing factors of the maternal microbiota have always been highly concerned. Although the abundance of many kinds of microbes has been proved to change significantly during pregnancy, the function of the specific microbes and their interaction with the host still need to be studied in depth. A precise understanding of the factors that affect the maternal microbiota is helpful to formulate an individualized plan, which can promote better management of the health of the mother and fetus during pregnancy.

### 2.3 The Role of the Maternal Gut Microbiota on Prenatal Development

The maternal nutritional status, immunity, and metabolism have always been the focus and difficulty in the study of the maternal-fetal interface. Maternal vital substances reach the placenta through blood circulation, and then spread or transport into the fetus’s body (15). During the first two-thirds of pregnancy, the mother exhibits hyperappetite and fat accumulation, at this time the development of the fetus is very limited (16). The fetus develops rapidly during the last third of gestation, when the maternal metabolism increases (16). The maternal microbiota are an important regulator of the metabolism and immunity during pregnancy, among which the gut microbiota are the most prominent (17–19). The gut microbiota can digest carbohydrates in the gut to produce vitamins, amino acids, and short-chain fatty acids (SCFAs) (20). As we all know, vitamins and amino acids are essential for fetal development, and SCFAs are also closely related to immunity and metabolism (21). Although the immune response and metabolic regulation mechanisms of adults are different from those of fetuses, it is also meaningful to explore the role of the maternal microbiota products in fetal development. The synthesis of SCFAs increases during pregnancy, which is necessary for the differentiation of regulatory T cells in the thymus of the fetus. In 2020, a study revealed that the changes in the diet of pregnant mice affect the growth and development of the fetus through the gut microbes (22). Specifically, pregnant mice fed undernourished (UN) or high-fat diet had lower plasma folic acid concentrations compared with the control group, and the relative abundance of the three *Lactobacilli* taxa in the gut of pregnant mice on a high-fat diet was higher compared with the control group. UN-fed or high-fat diet pregnant mice exhibit metabolic dysfunction and the fetal growth restriction. Furthermore, several studies suggested that the specific microbes in the maternal intestine are necessary for fetal immune system and neurodevelopment (3, 23, 24). The studies pointed out that segmented filamentous bacteria (SFB, common commensal bacteria in the cecum and ileum of mice) in the maternal intestine is the determinant of maintaining the level of IL-17A (IL-17A is related to the maternal immune activation). Consistently, the lack of IL-17A protects the fetus from behavioral disorders and neurodevelopmental disorders.

In summary, we conclude that the maternal gut microbiota and its metabolites are closely associated with the prenatal development of the fetus. However, we did not introduce the role of the maternal vagina and other parts of the microbiota in prenatal development because the current evidence in these areas is limited.

### 2.4 The Maternal Microbiota and APOs

Complications during pregnancy and APOs are the two most closely related to the maternal microbiota. We have previously introduced the former (14); here, we continue to introduce the latter. Currently, the APOs associated with the maternal microbiota include late abortion (LA), preterm rupture of membranes (PROM), hyperemesis gravidarum (HG), premature delivery (PTD), intrauterine growth restriction, and stillbirth (25). Worldwide, PTD is the most important factor leading to neonatal death (26). Also, intrauterine infection accounts for about 25%–40% of PTD (27). However, the cause of PTD caused by the maternal microbiota has not yet been revealed. The direct sources of the maternal microbiota that can cause fetal infection include placenta, umbilical cord, amniotic fluid, and fetal membranes; indirect sources include the gastrointestinal tract, vagina, skin and mucous membranes.

#### 2.4.1 The Uterine Microbiota and APOs

The serious consequence of the bacterial invasion of the amniotic cavity is neonatal death (26). The uterine microbiota are minute and important (28). Bacterial culture experiments indicated that positive bacterial culture in amniotic fluid is positively correlated with gestational age, and the microbial composition of the first and third trimesters is different (29). The microbial load in the amniotic cavity of pregnant women with premature rupture of membranes is related to the severity of the inflammatory reaction in the amniotic membrane (30). The frequency of the microbiota in the amniotic cavity of PTD women is 40%, and 29% of them have sterile intraamniotic inflammation (31). The level of proinflammatory cytokines in the amniotic fluid of women who give birth within 34 weeks of gestation is upregulated (the full-age gestation period is approximately 280 days), which links PTD to intraamniotic infection (32, 33). Further research revealed that there are uncultivated microbes that may induce inflammatory response in the amniotic fluid of PTD women. In addition, culture-independent techniques showed 46% of women with PTD have intrauterine infections (34). The development of detection technology has increased the detection rate of microbes in amniotic fluid, which promotes the understanding of the relationship between the uterine microbiota and APOs. Currently, the clinical significance of PCR-positive amniotic fluid seems to be consistent with that of positive cultures (35). However, the mechanism by which the uterine microbes cause APOs, especially PTD, is still unclear.

#### 2.4.2 The Vaginal Microbiota and APOs

The vaginal microbiota change not only during the female menstrual cycle but also during the reproductive process (36). The vaginal microbiota are closely related to women’s health, especially APOs. Malnourished women have an increased inflammatory response to the vaginal microbiota, which would lead to spontaneous preterm delivery (sPTD) (37, 38). *Lactobacillus* is the dominant species in the vagina, and its level may affect the APOs of pregnant women. Pregnant women with the low level of *Lactobacilli* in the vagina exhibit an increased risk of sPTD and diversity of *Mycoplasma* and *Gardnerella vaginalis* (38–40). Consistent with this, women with term deliveries have high levels of *Lactobacillus* and low bacterial diversity in the vagina (41, 42). However, not all *Lactobacillus* species in vagina are beneficial for pregnancy outcomes. For example, *Lactobacillus iners* is a risk factor leading to sPTD, but *Lactobacillus crispatus* is protective against sPTD (41, 42). A study in which participants were mainly African Americans showed that the diversity and abundance of vaginal microbes in term delivery women are significantly higher than that in PTD women (42). Concretely, *Bifidobacterium longum*/*Bifidobacterium breve*, *Lactobacillus gasseri*/*Lactobacillus johnsonii*, *Lactobacillus iners*/*Ralstonia solanacearum*, and *Lactobacillus crispatus*/*Lactobacillus acidophilus* of the vaginal microbiota might be related to decreased risk of PTD, and the vaginal community state type not dominated by *Lactobacillus* might be related to increased risk of PTD (43). In addition, the increased risk of PROM is associated with aerobic vaginitis (*p* < 0.05) (44). Consistently, Naomi Juliana et al. found that in Nigerian pregnant women, bacterial vaginosis is not associated with miscarriage and intrauterine growth retardation, but is associated with PROM, PTD, and low birth weight (45).

#### 2.4.3 The Gut Microbiota and APOs

Among the maternal microbiota, the differences in the gut microbiota are the most striking and influential. However, the studies on the relationship between gut microbiota and APOs are limited, and most of the studies focus on gut microbiota and pregnancy complications. It seems that the gut microbiota is difficult to link directly to APOs because the intestine and the fetus are far apart in human anatomy. In recent years, the colonization of gut microbes in the vagina has linked the maternal gut microbiota with APOs. The migration of *Listeria monocytogenes* from the intestine to the vagina, leading to maternal-fetal infection, is a good example (46). A Danish report revealed that *Listeria monocytogenes* in pregnancy pose a serious threat to the fetus, and the rate of miscarriage or stillbirth among mothers infected with *Listeria monocytogenes* is as high as 32% (46). In addition, pregnant women have multiple identical microbiota in their vaginas and rectum, and 68% of them have the same genotype (47). From here, we can see that to a large extent there is a corresponding relationship between the vaginal microbiota and the rectal microbiota. The above evidence supports the link between the gut microbiota and APOs through the vaginal microbiota. Therefore, the gut microbiota can be used as a reservoir for vaginal probiotics, and regulating the gut microbiota is an effective strategy for improving pregnant women’s APOs.

Numerous microbial metabolites and components in the intestine have a powerful ability to regulate inflammatory response and immunity, which provides another link between maternal gut microbiota and APOs (21). Evidence indicated that the relative abundance of *Prevotellaceae_UCG_003, Prevotella_1*, and *Selenomonas_1* in feces of aborted women is significantly reduced, and the level of proinflammatory cytokines in serum is increased (48). The study further pointed out that the metabolites of the gut microbiota are meaningful for recurrent miscarriage (48). Besides, the decrease in the alpha diversity of the gut microbiota is closely associated with the occurrence of sPTD, which may be regulated by the metabolites of the gut microbiota (49).

#### 2.4.4 The Placental Microbiota and APOs

The existence of the placental microbiota is controversial. Some scholars believe that the human placental microbiota is unique and similar to the composition of oral microbiota (11, 12). Sweeney et al. observed that *Ureaplasma* species are the most common microbes in the placenta, and their relative abundance is related to PTD and term delivery (50, 51). In contrast, a study of 76 term delivery does not support the presence of placental microbiota (52). The study pointed out that the occasionally observed microbes may be caused by pollution, which does not represent the presence of placental microbes. Coincidentally, Kuperman et al. found that the fetus in the womb is sterile through the study of 28 human placenta and six mouse placenta (53). They believe even if the placental microbiota are present, their biomass is extremely low and the impact on the fetus may be small. There is no proof which view is right at present. The studies of placental microbiota should be carefully designed, analyzed, and conducted in an unbiased manner.

#### 2.4.5 The Oral Microbiota and APOs

There is a Chinese proverb called “disease comes from the mouth”, which reflects the importance of oral microbes in human health from the side. There are many kinds of microbes that promote potentially APOs in the oral cavity, such as *Campylobacter rectus*, *Porphyromonas gingivalis*, *Fusobacterium nucleatum*, and *Filifactor alocis*. The pathways of the oral microbiota causing APOs may be (54): (1) Blood-borne spread of the oral microbes. (2) The immune response of the host and/or fetus to the pathogen causes inflammatory mediators to enter bloodstream. (3) Sexual behavior leads to the exchange of the oral and vaginal microbes. *Porphyromonas gingivalis*, the culprit of periodontitis, is a common anaerobic bacteria in the oral cavity and associated with many kinds of APOs, including fetal growth restriction (55), spontaneous abortion (56), and PTD (57). The mechanisms of *Porphyromonas gingivalis* causing APOs may be as follows: (1) *Porphyromonas gingivalis* directly invades the maternal tissue or placenta at the maternal-fetal interface (58). (2) *Porphyromonas gingivalis* evades the killing of the immune system by suppressing the immune response in the maternal tissues (59, 60). (3) *Porphyromonas gingivalis* leads to increased inflammatory response in maternal and fetal tissues *via* regulating the immune response (61). (4) *Porphyromonas gingivalis* promotes the increase of acute-phase proteins in maternal and fetal tissues, such as alpha-fetoprotein and C-reactive protein (62). (5) *Porphyromonas gingivalis* promotes the invasion and colonization of other maternal microbes. Therefore, the infection of *Porphyromonas gingivalis* could cause a systemic reaction to result in APOs, which is extremely detrimental to the health of the mother and fetus. It is very important for pregnant women to have good oral hygiene by controlling the oral microbiota.

## 3 The Microbiota and Offspring During Perinatal Period and Early Life

From birth to adulthood, environmental factors interact with genetic factors to maintain the healthy growth of the individual. During perinatal period and early life, the microbiota of offspring is closely related to the maternal microbiota, especially the neonatal and infant gut microbiota. Perinatal period and early life are the prime time for the colonization of the gut microbiota of offspring. The mode of delivery and gestational age at birth are important factors affecting the neonatal gut microbiota during the perinatal period. In early life, the determinants of the infant gut microbiota include feeding mode, maternal diet, environmental factors, and host genotype. In the following section, we introduce the establishment and development of neonatal gut microbiota and the driving forces of neonatal and infant gut microbiota.

### 3.1 Establishment and Development of Neonatal Microbiota

**Figure 2:**
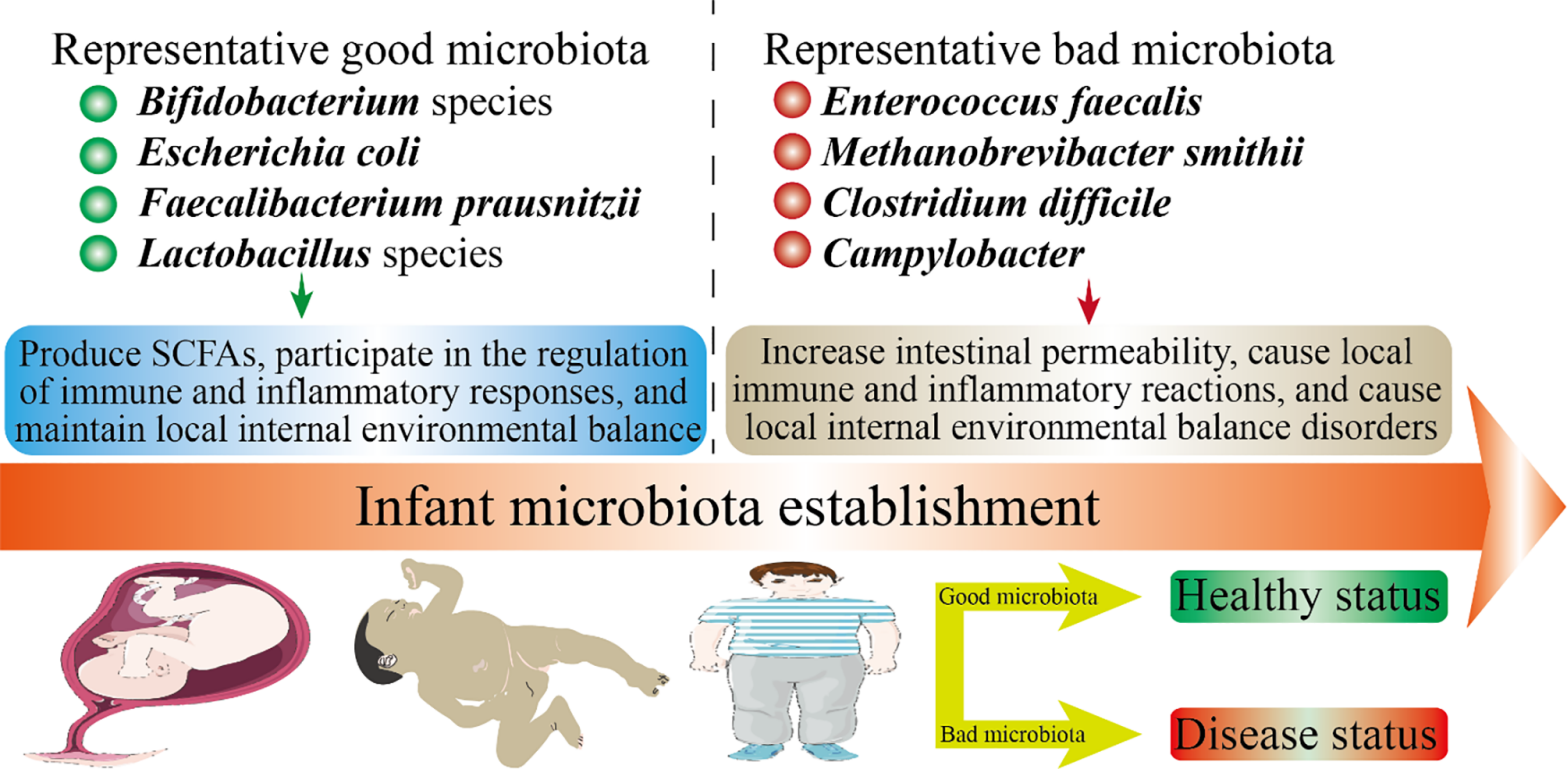
Infant health status and microbiota establishment. The health of offspring is closely related to the microbiota. In this figure, we have listed several representative good microbiota and several representative bad microbiota. The microbiota will inhibit the developmental stages throughout the offspring. The mechanism by which the good microbiota is beneficial to health may be: produce SCFAs, participate in the regulation of immune and inflammatory responses, and maintain local internal environmental balance. The mechanism by which the bad microbiota is harmful to health may be: increase intestinal permeability, cause local immune and inflammatory reactions, and cause local internal environmental balance disorders.

The establishment of neonatal gut microbes is the result of the interaction of environmental and host factors. Both beneficial and harmful microbes would colonize the neonatal intestines at birth and are profound for the health of infant (63) (**Figure 2**). The microbes that newborns are exposed to at birth are very important, especially the maternal microbes (64). Bacteria from the maternal vagina and skin have been observed in the feces of newborns (65). The mouth, skin, and intestines of newborns delivered vaginally are rich in *Lactobacillus* which is the core of the maternal vaginal microbiota (66). After birth, newborns would continue to obtain microbes from the maternal microbiota in different parts. Also, the colonization of these microbes is durable and stable.

The infant gut microbiota exhibits initially low diversity and then increases with early development. In early life, the microbes that colonize the intestine are usually aerobic bacteria because the intestine is aerobic. After adulthood, the microbes colonized in early life will be replaced by typical anaerobic bacteria in the intestine (67). Studies on the gut microbiota of infants indicated that *Clostridia*, *Enterobacteria*, and *Streptococci* were observed in the infant intestines in the first 2 days after birth (68). Clostridia, *Bacteroides*, and *Bifidobacterium* appear in the intestines of 40% of infant on the third day (68). On the 4th–7th days, *Bifidobacterium* became dominant, and its relative abundance is highest (68). Before weaning, the relative abundance of *Bacteroides* gradually increases to compete for *Bifidobacterium* in the infant intestine. After ingesting solid food, the composition of the infant gut microbiota is similar to that of adults (67). In 2–3 years after birth, the infant gut microbiota gradually develops towards the adult gut microbiota (66, 67).

### 3.2 The Driving Forces of the Infant Microbiota

The process from colonization to maturity of the gut microbiota is nonrandom and dynamic (69, 70). The process is affected by many factors during the perinatal period and early life, although it is difficult to quantify.

#### 3.2.1 The Mode of Delivery

The mode of delivery is a crucial factor affecting vertical transmission (71). The composition of the gut microbiota of the infant delivered vaginally appears to be similar to that of the maternal vaginal microbiota: *Lactobacillus* dominates, followed by *Senathia* spp. and *Prevotella*. While the gut microbiota of infant delivered by cesarean section is similar to that of the maternal skin: *Staphylococcus* is dominant, followed by *Propionibacterium* and *Corynebacterium* (65, 72). Cesarean section leads to the imbalance of infant gut microbiota and the decrease of the diversity (73, 74). There was a study: researchers exposed infant delivered by cesarean section to vaginal fluid to promote the recovery of the infant gut microbiota. Interestingly, this approach effectively improves the oral, gut, and skin microbiota of infant delivered by cesarean section within 30 days after birth (75). This indicates that the vertical transmission of the maternal vaginal microbes can be reversed to some extent after birth.

It is worth noting that epidemiology has linked cesarean section to autoimmune diseases, asthma, obesity, and allergies (76). The difference in infant microbiota caused by vaginal delivery and cesarean section may be attributed to the “bacterial baptism” of vaginal fluid. Vaginal microbes spread vertically to the infant to promote the colonization of the infant microbes. However, there is another voice here: the differences in the infant microbiota caused by cesarean section may due to the use of antibiotics during delivery, the inactivity of the mother, and complications during pregnancy (77). The current studies may overestimate the effect of vaginal delivery on infant microbiota. Furthermore, the difference in infant microbiota caused by cesarean section is more moderate in rodents compared with humans. Of course, this may be caused by the spread of microbes in rodents more easily than in humans. Therefore, convincing evidence is necessary to clarify the differences in infant gut microbiota caused by the mode of delivery.

#### 3.2.2 The Gestational Age at Birth

Gestational age at birth is also important for the colonization of infant microbiota. The immature microbiota poses a huge threat to premature because their immune and nervous systems are not yet fully developed. Usually, premature need to stay in the hospital for treatment. Factors such as the use of antibiotics and parenteral feeding can interfere with the natural establishment of infant microbiota. Compared with term infant, the intestines of premature cannot colonize symbiotic anaerobic bacteria such as *Bacteroides* and *Bifidobacterium*, and the relative abundance of *Enterococcus* and *Enterobacteriaceae* in their feces is increased (78–83). Studies showed that the gut microbiota of very low birth weight infant exhibits a transition from *Bacilli* to *Gammaproteobacteria* to *Clostridia* (84). The interaction between the immune system of premature infants and their immature microbiota may lead to inflammation and infectious diseases (85, 86). Compared with term infant, the SCFAs of the gut microbiota of preterm infant are significantly lower (81, 87). In short, the gestational age at birth affects the infant short-term health and even long-term health to a certain extent.

#### 3.2.3 The Feeding Mode and Maternal Diet

The feeding mode is mainly associated with the infant gastrointestinal microbiota. The role of breastfeeding in regulating the gut microbiota of infant has been widely recognized (88, 89). In particular, the relative abundance of *Bifidobacterium* in the intestines of the breastfed infant is increased (90). On the one hand, breastfeeding provides infant with maternal microbes, nutrients, and antibacterial agents, which are important for infant health. On the other hand, the IgA in breast milk contributes to the “tolerability” and “regulation” of the infant immune system (91). It should be emphasized that human milk oligosaccharides (HMOs) in breast milk can selectively shape the formation of beneficial microbes in infant. The bioactive molecules and identified microbes in breast milk are shown in **Table 2** (92–98). The gut microbiota of breast-fed infant is dominated by *Bifidobacterium*, while formula-fed is dominated by *Bacteroides* and *Bifidobacterium* (99). Besides, breastfeeding can effectively prevent infant from being overweight by improving the imbalance of the gut microbiota compared with formula-fed (100). Therefore, breast milk is the best food for babies. These also reflect the importance of infant microbiota in early health, and its impact on long-term health is also profound (101).

**Table 2:** Bioactive molecules and identified microbes in breast milk.

Category	Specific type
Identified microbes	Staphylococci, Streptococci, Corynebacteria, Propionibacteria, *Lactobacillus* spp., *Bifidobacterium* spp.
Anti-microbials	Immunoglobulins- Secretory IgA, IgM, IgG, lactoferrin, lactadherin/MFG E8, Lysozyme, complement C3, antiviral mucins—MUC1, MUC4
Digestive enzymes	Amylase, Bile acid-stimulating esterase, bile acid-stimulating lipases, lipoprotein lipase
Growth factors	Epidermal growth factor (EGF), nerve growth factor (NGF), insulin-like growth factor (IGF), transforming growth factor (TGF), taurine, polyamines, heparin-binding EGF like growth factor (HB-EGF), vascular endothelial growth factor (VEGF), erythropoietin
Transporters	Lactoferrin, folate binder, cobalamin binder, IGF binder, thyroxine binder, corticosteroid binder
Hormones	Calcitonin, somatostatin, adiponectin, leptin, ghrelin
Oligosaccharides and glycans	Human milk oligosaccharides (HMOs), gangliosides, glycosaminoglycans
Cytokines, chemokines, and anti-inflammatory factors	Tumor necrosis factor-alpha (TNF-α), interferon-gamma (IFN-γ), Transforming growth factor-beta (TGF-β), prostaglandins, 1-antichymotrypsin, 1-antitrypsin, platelet-activating factor: acetyl hydrolase, interleukins-IL-6, IL-7, IL-8, IL-10 chemokines-granulocyte colony stimulating factor (G-CSF), macrophage migratory inhibitory factor (MIF)

The maternal diet may affect the baby’s gut microbes through milk. Milk can be regarded as a kind of body fluid, and the maternal diet affects directly the microbial composition and diversity of breast milk (102, 103). During lactation, maternal fat consumption and fiber intake are important determinants of breast milk microbiota (104). Furthermore, maternal diet also affects the bioactive molecules in milk, such as HMOs (105). Therefore, breastfeeding mothers should diet carefully to give the best care to the baby.

#### 3.2.4 The Environmental Factors

It is also meaningful to study the relationship between environmental factors and the colonization of microbiota in early life. Despite the lack of in-depth research, family members have been defined as potentially affecting the colonization of infant gut microbiota (63, 106). Interestingly, a Dutch study showed that the relative abundance of *Bifidobacterium* in the gut microbiota of infant with siblings (1 month old) is higher than that of those without siblings (107). In addition, the level of facultative anaerobes and anaerobes in the infant gut without big sisters is lower, while that of *Clostridium* and *Escherichia coli* is higher (108). A Danish study showed that the abundance and diversity of gut microbes of infant with siblings in early life are increased, while the influence of pets on them was not obvious (109). However, the concept of the “sibling effect” remains controversial because it is so extensive and difficult to quantify. Geographical location may also affect indirectly the microbiota in early life because geographic location determines eating habits and lifestyle (63, 110). For example, the diversity of infant microbiota living in rural Africa is significantly different from that of in cities (111).

#### 3.2.5 Host Genetics

In recent years, the studies on host genetics and infant gut microbiota have gradually increased (112, 113). The most convincing evidence is that the microbial similarity of children with genetically identical twins (under 10 years of age) is higher than that of fraternal twins and unrelated controls (114). Additionally, the relative abundance of *Bifidobacterium* is associated with single nucleotide polymorphisms (SNPs) located at the LCT site of human genes (human lactase gene) (115). However, genetic factors are complex, and more evidence is needed to describe its association with the microbiota in early life.

## 4 Infant Gut Microbiota

In **Table 3**, we summarize the changes in the microbial composition of some parts of the infant body from delivery to postpartum (116). In early life, the infant gut microbiota undergo a transition from being dominated by *Lactobacillus* and *Bifidobacterium* to being dominated by *Firmicutes* and bacteria (117), which represents the gut microbiota from infancy to maturity. *Lactobacillus* and *Bifidobacterium* can promote the development of infant-acquired immunity and innate immunity in early life. Importantly, the relative abundance of *Bifidobacterium* in feces in early life is related to the risk of developing noncommunicable diseases in later life, such as obesity and asthma (118, 119). The composition and diversity of infant gut microbiota change dramatically with the intake of solid foods. The dietary fiber fermented by the gut microbiota produces SCFAs, which are related to the host’s immunity and metabolism (21, 120). Below, we introduce in detail the gut microbiota of infant.

**Table 3:** Changes in the microbial composition of various parts of the infant body from delivery to postpartum.

Microbiota\sites	Gastrointestinal tract	Skin	Oral mucosa	Nares
Actinobacteria	↓↓	↑	↓↓	↑
Bacteroidetes	↑↑	↓	↑↑	↑
Firmicutes	↑	→	↑↑	↓
Fusobacteria	↓	↓↓	↓	↑
Proteobacteria	↓	↓	↓↓	↑↑
Others	↓	↓↓	↓	↑

“↑“ represents an increase in the abundance of the microbe from delivery to postpartum. “↑↑“ represents a significant increase in the abundance of the microbe from delivery to postpartum. “↓“ represents a decrease in the abundance of the microbe from delivery to postpartum. “↓↓“ represents a significant decrease in the abundance of the microbe from delivery to postpartum. “→“ represents that the abundance of the microbe is basically unchanged from delivery to postpartum.

### 4.1 The Dominant Populations of Infant Gut Microbiota

The adult gut microbiota are relatively stable (121), while the infant (less than 1 year old) have a lower diversity of gut microbiota compared with other age groups (110). Infant gut microbiota are dominated by *Bifidobacterium*, but there is great variability among individuals. According to the dominant population and composition, the core of infant gut microbiota is mainly divided into six groups (122): group 1, *Bifidobacteriales*, *Lactobacillales*, *Anaerostipes*, *Clostridiales*, and *Faecalibacterium*; group 2, *Verrucomicrobiales* and *Bacteroidales*; group 3, *Clostridiales*; group 4, *Enterobacteriales*; group 5, *Pasteurellales*; and group 6, *Selenomonadales*. In **Table 4**, we summarize the dominant populations and their functions of the infant gut microbiota.

**Table 4:** The dominant populations of infant gut microbiota.

Microbes	Key description	Refs.
Bifidobacteria	Bifidobacteria make a major metabolic contribution to their host through the degradation of diet-derived glycans and host-provided carbohydrates (known as host glycans and including mucins and HMOs).	(123)
*Clostridia* class	They are known as pathogenic microorganisms that may cause bacteremia and pseudomembranous colitis, and their presence at high densities is interpreted as an indicator of an unhealthy microbiota.	(124)
Genus *Bacteroides*	Members of this genus are classified as saccharoclastic bacteria that are able to metabolize host-produced glycans, such as HMOs and mucins, but also complex plant polysaccharides such as starch, cellulose, xylans, and pectins.	(125, 126)
Genera *Veillonella*	These bacteria are saccharolytic and utilize end products of carbohydrate fermentation (e.g., lactate) of other infant gut bacteria, such as Streptococcus spp. and Bifidobacterium spp., to produce propionate, forming an important trophic chain.	(127)
Genera *Streptococcus*	They are among the first established bacteria in the infant gut, where they can be identified within the first 24 h following birth.	(128)
Genus *Collinsella*	Members of the genus *Collinsella* have recently been shown to reach high numbers when they are associated with an infant gut microbiota dominated by bifidobacteria.	(129)
Genus *Lactobacillus*	Vertical transmission of *Lactobacillus* species presents the origin of infant Lactobacillus microbiota component. And they may be related to the HMO metabolism of infants.	(128)
Genus *Akkermansia*	They can provide a barrier for the baby’s intestines and may participate in the fermentation of HMO.	(130)

#### 4.1.1 Bifidobacteria

*Bifidobacteria*, belonging to the phylum Actinomycetes, anaerobic gram-positive bacilli, was isolated from the feces of the breastfed infant as early as 1899. At present, *Bifidobacteria* have been widely used in medicine and food. New strains are constantly being developed. At present, the ones that have been found to be closely related to infant health are *Bifidobacterium breve* and *Bifidobacterium longum*. The vertical transmission of the maternal *Bifidobacterium* to the offspring promotes its distribution in nature. The identification experiments of common *Bifidobacterium* in mothers and offspring confirmed this conclusion (69, 131).

HMOs in breast milk are natural prebiotics, and their absorption in the intestinal tract of infant mainly depends on *Bifidobacteria* (132). The evidence is that the level of HMOs in the feces of infant is negatively correlated with the level of *Bifidobacteria*. Interestingly, HMOs have no direct nutritional value for infant, and their important function is to shape the infant gut microbiota and benefit long-term health (133). After premature infant are supplemented with *Bifidobacterium longum*, the inflammatory response is weakened and the intestinal permeability is reduced (133). Premature infants supplemented with *Bifidobacteria*-based microbes can restore the gut microbiota to the level of term infant (134). Therefore, in early life, Bifidobacterium can effectively improve the health of infant, whether they are the premature infant or term infant.

#### 4.1.2 The Genus *Lactobacillus*

The genus *Lactobacillus* are also known to be the dominant species in the infant gut microbiota and observed shortly after delivery (128). Various genus *Lactobacillus* are detected in meconium (the relative abundance: vaginal delivery is significantly greater than caesarean section), including *Lactobacillus reuteri*, *Lactobacillus plantarum*, *Lactobacillus sakei*, *Lactobacillus brevis species*, and *Lactobacillus casei* (135). Studies on the gut microbiota of infant in early life suggested that *Lactobacillus gasseri* and *Lactobacillus rhamnosus* are dominant in terms of the genus *Lactobacillus* (136). In addition to being abundantly present in the infant gut microbiota, the mother’s vagina and milk are also rich in the genus *Lactobacillus*. The former is passed vertically to the offspring during vaginal delivery and the latter is passed to the offspring during breastfeeding. The main types of the genus *Lactobacillus* in milk are *L. plantarum* and *L. pentosus* (137). As mentioned above, apart from *Bifidobacteria*, the genus *Lactobacillus* can also digest HMOs, and there are obvious differences in the ability of different strains to ferment HMOs (138). Furthermore, *Lactobacillus reuteri* DSM17938 has been shown to be effective in relieving colic of breast-fed infant (139).

#### 4.1.3 The Genus *Clostridium*

The genus *Clostridium* can be divided into multiple genera, all of which belong to the *Clostridia* class. The genus *Clostridium* is the only genus of anaerobic bacillus, and their spores are round or ovoid, their diameter is wider than the bacteria. Most *Clostridium* species in the intestines of infants are pathogenic, especially *Clostridium perfringens*, *Clostridium difficile*, *Clostridium tetani*, and *Clostridium botulinum* (140). *Clostridium* species are common settler in infant intestine, but they are usually asymptomatic (141). The results of infant fecal microbial culture showed that the level of *Clostridium perfringens* and other *clostridia* may reach 107 CFU/g (142). A stool study of infant (<1 year old) in Jordan indicated that the colonization rate of *Clostridium perfringens* in the infant intestines is 27.2%, and the colonization rate of infant <6 months is higher (*p* < 0.004) (143). Interestingly, Rada et al. found that both the *Clostridia* and *Bifidobacteria* in the feces of infant (3–253Radadays old) grow vigorously on the prebiotic oligosaccharides, which may pose a challenge for supplementing prebiotics to infant lacking *Bifidobacteria* (144). In contrast, *Enterococcus faecalis* isolated from infant feces can inhibit the growth of *Clostridium difficile*, which may have potential applications for preventing *Clostridium difficile* colonization and infection (145). In short, in early life, we need to beware of *Clostridium* to prevent them from becoming the dominant species in infant gut microbiota.

However, Kim et al. found that neonatal acquisition of *Clostridia* species protects against colonization by bacterial pathogens (146). The article pointed out that the administration of *Clostridiales* enhances the colonization resistance of the infant intestine, which can prevent some pathogens from attacking the infant intestine. Consistent with this, commensal *Clostridia* is considered to play an important role in maintaining intestinal homeostasis (147, 148). These data improve our understanding of the function of infant microbes. The application of *Clostridia* species to regulate the homeostasis of the infant intestinal microbiota is still a huge challenge, because most *Clostridia* are pathogenic.

#### 4.1.4 The Genus *Bacteroides*

From birth to adulthood, the human microbiota gradually mature, among which the infant microbiota are usually characterized by a low total level. The relative abundance of *Bacteroides* in the infant feces delivered vaginally is higher than that of cesarean section (*p* < 0.01) (149). The diversity of *Bacteroides* in the feces of formula-fed infant is higher than that of breast-fed infant (150). Furthermore, the delayed colonization of *Bacteroides* caused by cesarean section may be associated with Th1 response (151). Similar to *Bifidobacteria* and the genus *Lactobacillus*, *Bacteroides*, and *Fragilis* in infant intestine can also digest HMOs (125). Additionally, in the environment of carbohydrates and HMOs, *Bacteroides thetaiotaomicron* promotes the growth of bacteria that produce SCFAs (152). However, some *Bacteroides* are pathogenic. For example, *Bacteroides fragilis* can cause anaerobic meningitis in infant (153). The abundance of *Bacteroides* in the intestine of allergic infant (<2 years old) is higher than that of allergic infant (154).

#### 4.1.5 The Genus Veillonella and Streptococcus

The genus *Veillonella* are strictly anaerobic and parasitizes in the mouth, intestine, and respiratory tract of humans and animals (155). They can produce endotoxins and therefore play a role in various mixed infections. They are often detected from soft tissue abscesses and blood and are usually normal microbiota (155). The genus *Veillonella* is also common in the intestine of infants. In recent years, the new species have been identified continuously. For example, in 2018, Mashima et al. isolated a new type of anaerobic Gram-negative cocci strain from the biofilm of children’s tongue (156). Later, they found that the strain is different from the previous *Veillonella* species through genetic sequencing, and named it *Veillonella infantium*. An interesting study showed that the relative abundance of *Veillonella* in the intestine of infant (2–6 months old) exposed to unsafe food caused by Hurricane Maria is reduced (157).

The genus *Streptococcus* is another major group of common Gram-positive cocci in pyogenic cocci. There are 69 species and subspecies, which are widely distributed in nature and the nasopharynx, gastrointestinal tract of the human body, and most of them belong to normal genus (158). Pathogenic *Streptococci* can cause a variety of purulent inflammation and hypersensitivity diseases in humans, among which group B *Streptococcus* (GBS) are the most threatening to infants (159). Although antibiotics can reduce GBS level during delivery, GBS is still an important cause of neonatal sepsis in some high-income contexts (160). Approximately 35% of pregnant women carry GBS, and the gut microbiota of the offspring of mothers who carry GBS are significantly different from those of mothers who do not carry GBS (161). Therefore, effective prevention of the colonization of the GBS is essential for infant health.

#### 4.1.6 The Genus Collinsella and Akkermansia

In the infant gut microbiota dominated by *Bifidobacteria*, the level of the genus *Collinsella* can be very high (129). So far, the genus *Collins* includes five species, all isolated from the human gastrointestinal tract: *Collinsella stercoris*, *Collinsella intestinalis*, *Collinsella aerofaciens*, *Collinsella tanakaei*, and *Collinsella ihuae* (162–164). *Akkermansia muciniphila* is the only specie of the genus *Akkermansia* in the gut microbiota, although its relative abundance is very low (165, 166). The level of *Akkermansia muciniphila* in the intestine rises rapidly after weaning and increases with age (167). Animal experiments indicated that the abundance of *Akkermansia muciniphila* in the intestine of obese and type 2 diabetic mice is reduced, and administration of *Akkermansia muciniphila* can control the inflammation and reduce intestinal permeability (168). Notably, *Akkermansia muciniphila* has also been found to be involved in the fermentation of HMOs.

## 5 The Role of Infant Microbiota in Future Health Planning

From birth, the gut microbiota are responsible for the activation and development of the immune system, the development of the central nervous system (CNS), and the digestion and metabolism of food. Early life is a critical period for microbial colonization, which not only affects the health of infant, but also is profound to long-term health planning (**Figure 2**). Below, we introduce in detail the relationship between infant gut microbiota and brain development and the shaping of the immune system. We also talk about the role of microbiota in early life in diseases and the direction of future study on human microbiota.

### 5.1 Infant Brain Development

**Figure 3:**
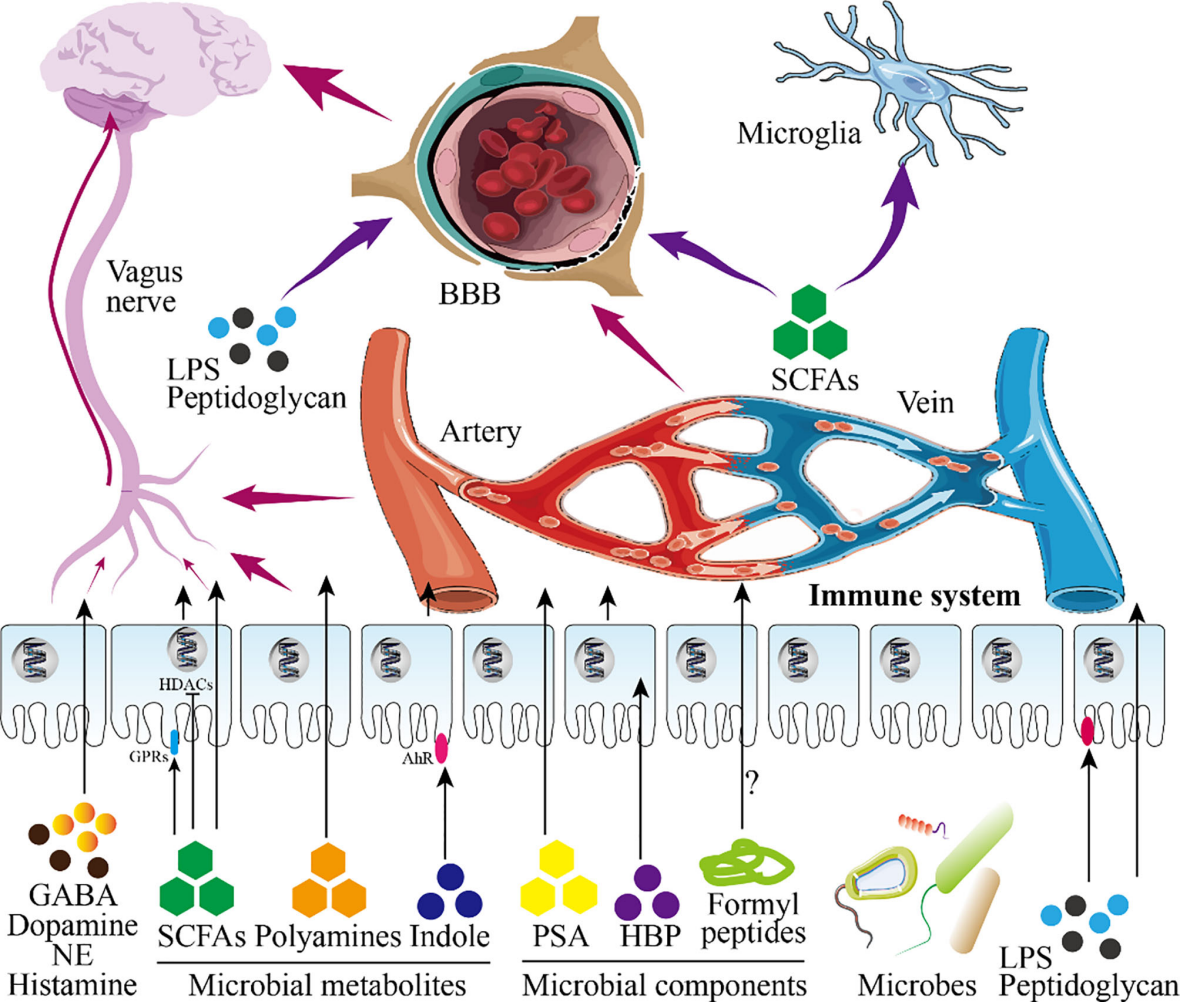
Infant gut microbiota and central nervous system. This figure shows the role of infant microbiota in the central nervous system (CNS). Microbial metabolites and microbial components directly pass-through epithelial cells or act on receptors on epithelial cells to play a role (in this figure, intestinal epithelial cells are taken as an example). On the one hand, these substances will further reach the blood-brain barrier (BBB) after entering the blood circulation and enter the brain to participate in the regulation of the CNS after passing through the BBB. On the other hand, these substances continue to undergo biochemical reactions in the blood circulation to affect the CNS. Besides, SCFAs and LPS entering the blood circulation can also directly act on nerve cells. Shortly, these substances regulate the development of the brain and CNS.

Early life (less than 2 years old) is the period of rapid brain development. The brain volume of newborns is only about 1/3 the size of adults, but their brains will increase to 80%–90% of adults by the age of 2 (169). In early life, the infant brain undergoes successively the growth of axons and dendrites, the formation of synapses, the expansion of neurogliocyte, and the myelination of axons. Importantly, the first 2 years of life is also a golden period for the establishment of the gut microbiota. Therefore, the establishment of the gut microbiota in early life is essential for the development of the infant brain (169). In **Figure 3**, we briefly describe the role of the gut microbiota in the development of the CNS early in life (**Figure 3**).

There is a Chinese proverb: pull one hair and the whole body is affected. This sentence well reflects the role of the gut microbiota in brain development. What must be mentioned here is the ability of the metabolites or components of the microbiota to promote the development of the nervous system. These important molecules can enter the blood circulation through the intestinal barrier to affect brain development (170). For example, the indigenous spores produced by bacteria promote the synthesis of serotonin in enterochromaffin cells (ECs), thereby regulating the serotonin levels in the circulation, colon and ileum (171). Besides, SCFAs, the product of dietary fiber fermented by the gut microbiota, can directly regulate the sympathetic nervous system through free fatty acid receptors (belonging to G protein-coupled receptors) (21, 172). Kimura et al. found that SCFAs produced by the pregnant mother determine the development of the fetal intestine, pancreas, and nerves through GPR43 and GPR41 (173). SCFAs can also promote the maturation of microglia which differentiate retrogradely into other types of glial cells (174). Furthermore, the permeability of the blood-brain barrier (BBB) of germ-free mice is increased, and the administration of sodium butyrate (a type of SCFAs) improves the permeability defect of the BBB of germ-free mice by inhibiting histone deacetylases (HDACs) (175). In addition, the diversity of the gut microbiota is related to the release of intestinal peptides (such as oxytocin, ghrelin, glucagon-like peptide, cholecystokinin) (176). The disorder of the maternal gut microbiota causes the fetal brain to fail to develop normally, which is mediated by the metabolites of maternal gut microbes (177). The insufficiency of the gut microbiota is a great threat to premature babies because it weakens the ability of the intestinal wall to resist pathogens (170).

On the basis of its influence on brain development, the relationship between the gut microbiota and behavior in early life has also been established in recent years. For example, there are data showing that the diversity of the gut microbiota is associated with the cognitive ability of infant (170). In short, gut microbiota can shape the individual behaviors and social behaviors of infant (178).

### 5.2 Infant Immune System Development

Before birth, the maternal microbiota participate in regulating the innate immunity of the fetus. After birth, the infant microbiota continue to shape innate immunity and acquired immunity to promote the development of the immune system to a normal state.

#### 5.2.1 Microbial Colonization Modulates Early Development of the Immune System in Mucosal Tissues

The microbiota on the mucosal surface in the early life changes dynamically until they reach an equilibrium point, then remain stable throughout long-term planning in the absence of external or internal insults (179). The colonization of microbes on the mucosal surface and the shaping of the immune system occur simultaneously in early life. Therefore, the microbiota on the mucosa may directly or indirectly shape the immune system until it matures. In **Table 5**, we summarize the gut microbes that have been identified in recent years to regulate intestinal mucosal immunity. The immune system of newborns is very different from that of adults, because infant preferentially develop tolerance in response to pathogen invasion (195–197). The period after birth is the most critical period, during which exposure to the microbiota may have a profound impact on the structure and function of the infant immune system.

**Table 5:** Identified gut microbes regulating intestinal mucosal immunity.

Microbial species	Involved substances	Involved intermediates	Key description	Refs.
*Lactobacilli*, *Clostridiales* members	Tryptophan indole derivatives	AhR	IL-22 production; resistance to enteric pathogens; maintenance of intestinal homeostasis and barrier functions	(180–182)
Various microbes including *Bacteroides* spp.	SCFAs	Receptors GPR41, GPR43 and GPR109, HDAC inhibition, mTOR, STAT3, ERK and MAPK signaling	Protective inflammatory responses during pathogen infection; secretion of AMPs, chemokines and cytokines; controls IECs turnover and barrier functions; RALDH1 expression and vitamin A metabolism	(21)
*Clostridium sporogenes*	Indole 3-propionic acid	PXR	Regulation of intestinal permeability and intestinal inflammation, defense against intracellular pathogens	(183)
*Clostridial* species	Mechanosensors/mechanotransducer Piezo2	Cellular forces	Serotonin release by enterochromaffin cells	(171, 184)
*Bacteroides vulgatus*, *Enterococcus faecium*	Nod2	Peptidoglycan components; muramyl dipeptide	Restriction of bacterial growth or dissemination, expression of inflammatory genes, goblet cell function	(185)
*C. rodentium, S. typhimurium, H. pylori*	Unmethylated CpG bacterial DNA	NF-κB, TLR9	Decreases intestinal inflammation and damage following bacterial challenge	(186)
*Helicobacter hepaticus, S. typhimurium*, *Pasteurellaceae* family	Autophagy	Cellular stresses	Control inflammation-induced apoptosis, necroptosis and maintains intestinal barrier, lysozyme secretion by Paneth cells, promotes bacterial clearance	(187)
*Rotavirus EW*	dsRNA	Nlrp9b inflammasome	Restricts rotavirus infection by IL-18 production and pyroptosis	(188)
Commensal gut microbes	Free fatty acids	TLR4, PPAR	Prevents development of metabolic syndrome; regulates expression of lysozyme and PPAR-controlled genes	(189)
C. rodentium	TLR ligands	MyD88 signalling	Secretion of AMPs, control of bacterial infiltration, enhanced barrier integrity	(190)
*Toxoplasma gondii*	ligands include extracellular ATP	P2X7R/NLRP3 inflammasome	IL-1β secretion and inhibition of parasitic proliferation	(191)
*Lactobacillus rhamnosus, Ruminococcus gnavus*	Pili	GPR and ERK/MAPK signaling	Expression of glycoroteins and mucus production by goblet cells; cytoprotective responses	(192)
*Salmonella Typhimurium, C. rodentium*	Flagellin	NAIP/NLRC4 inflammasome	Protects against enteric pathogen invasion; expulsion of pyroptotic IECs and release of eicosanoid and IL-18	(193)
*Clostridium difficile*	Enterotoxins	Caspase-3/7-mediated apoptosis	Restricts C. difficile growth *in vivo*	(194)

AhR, aryl hydrocarbon receptor; SCFAs, short-chain fatty acids; GPR, G protein-coupled receptor; PXR, pregnane X receptor; HDAC, histone deacetylase; RALDH1, aldehyde dehydrogenase 1; Nod2, nucleotidebinding oligomerization domain 2; TLR9, toll-like receptor 9; dsRNA, double-stranded RNA; Nlrp9b, NLR family, pyrin domain containing 9B; PPAR, peroxisome proliferator-activated receptor; P2X7R, P2X7 receptor; IECs, Intestinal epithelial cells.

#### 5.2.2 The Mechanisms of the Microbiota Regulating Immunity

##### 5.2.2.1 Microbial Metabolites

**Figure 4:**
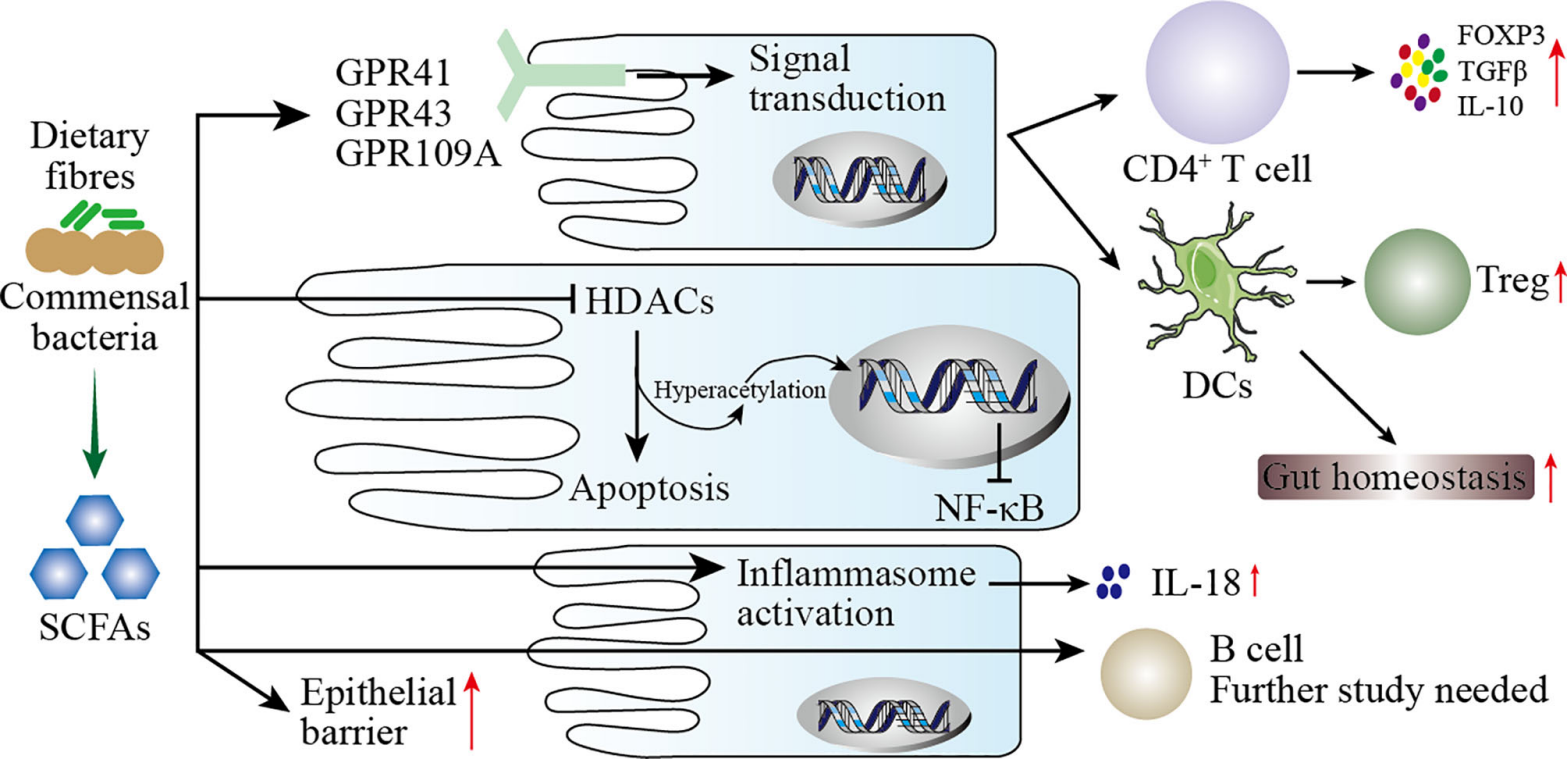
Mechanisms of SCFAs regulating mucosal immunity. SCFAs are produced by the digestion of dietary fiber by the intestinal microbiota. On the one hand, SCFAs increase the barrier function of the intestinal mucosa to protect the body from harmful microbes. On the other hand, SCFAs participate in the mucosal immunity of the intestine, specifically: (1) SCFAs act on G protein-coupled receptors (GPR) on intestinal epithelial cells (IECs) to activate downstream cell signal transduction to regulate the immune response of T cells and dendritic cells (DCs). (2) After SCFAs enter IECs, they act as histone deacetylases (HDACs) inhibitors to affect the transcription of inflammatory genes. (3) SCFAs activate inflammasomes in IECs to promote the secretion of IL-18. (4) SCFAs directly pass through IECs to regulate the immune response of B cells.

The metabolites of gut microbiota are diverse, and their sources include gut microbes digesting exogenous food components and fermenting endogenous compounds. These metabolites may act directly on the mucosa or enter the blood circulation through mucosal epithelial cells. The mechanism by which these metabolites modulate the immune response may be as follows (including but not limited to) (198): (1) Gut microbes digest dietary fiber to produce SCFAs which have wide-ranging effects on the immune system (**Figure 4**); (2) metabolites of gut microbes bind to specific receptors on host cells, including aryl hydrocarbon receptor (AHR), G protein-coupled receptors 41 and 43, Toll-like receptors (TLRs), and PXR receptors (see **Table 5**); (3) polyamines, such as spermine, putrescine, and spermidine, are involved in gene transcription and translation and exist in most living cells.

##### 5.2.2.2 Microbial Components

**Figure 5:**
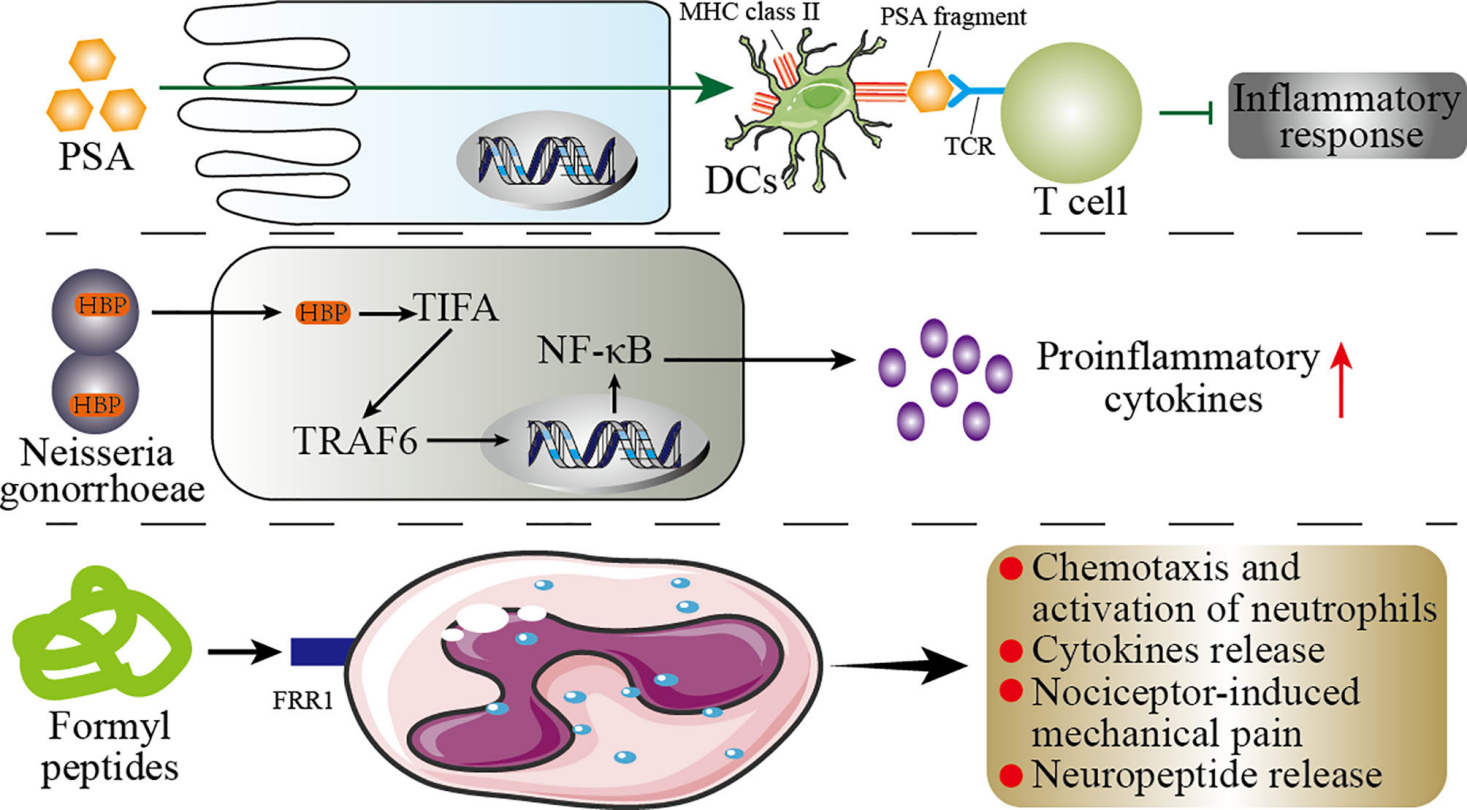
Mechanisms of the microbial components regulating immunity. This figure shows the role of the microbial components polysaccharide A (PSA), formyl peptide, and HBP (d-glycero-β-d-mannoheptose-1,7-bisphosphate) in the host immunity. PSA can enter the circulatory system directly through the host’s intestinal epithelial cells (IECs). In the circulatory system, PSA connects dendritic cells (DCs) and T cells through MHC class II and T-cell receptors (TCR) to inhibit inflammation. In the host’s genitourinary tract, HBP promotes the secretion of proinflammatory cytokines by the host’s immune cells through the TIFA/TRAF6/NF-κB pathway. Formyl peptide binds to neutrophils by binding to formyl peptide receptors 1 (FRR1) to regulate the inflammatory response of neutrophils.

Pattern recognition receptors (PRRs) are important components of innate immunity, which are responsible for responding, detecting, and coordinating self and nonself-antigens (198). PRRs can respond to the components of a variety of microbes (such as fungi, viruses, and bacteria), including peptidoglycans, lipopolysaccharides, formyl peptides, unique nucleic acid structures, and flagellin. The activation of PRRs promotes the release of chemokines, apoptotic factors, and cytokines through a signal cascade to participate in disease occurrence (199). We summarize briefly the mechanism by which microbial components modulate the immune response in **Figure 5**.

### 5.3 The Microbiota in Early Life as a Possible Predictor of the Human Health

From pregnancy to long-term planning, the microbiota plays an important role in the health of the baby. The normal microbiota of infant has a positive impact on the health in early life and long-term planning, while disturbance of the microbiota may be one of the causes of brain dysplasia, immune system defects, and metabolic disorders. In **Table 6**, we summarize the microbial metabolites or components related to diseases that have been identified in recent years. Therefore, the formation factors of the infant microbiota are essential for establishing targeted strategies to minimize the early interference and long-term effects on the human health (213).

**Table 6:** Microbial metabolites or components related to diseases.

Microbial metabolites or components		Human disease or preclinical models	Refs.
Cancer	Colorectal cancer	SCFAs, B vitamins, N1, N12-diacetylspermine	(200, 201)
Gynecological and reproductive disorders	Bacterial vaginosis and other sexually transmitted infections	Polyamines, HBP	(202)
	Preterm labor	SCFAs	(203)
Allergic and immune disorders	Asthma	SCFAs	(204, 205)
	Inflammatory bowel disease	SCFAs, B vitamins	(206)
Neurological disorders	Central nervous system dysfunction	SCFAs	(174)
	Autism spectrum disorder	4-EPS	(207)
Metabolic disorders	Obesity and metabolic syndrome	TMAO	(208)
	Kidney disease	p-Cresol, SCFAs	(209)
	Type 2 diabetes	TMAO	(210)
	Cardiovascular disease	TMAO	(209, 211)
Other gastrointestinal disorders	Infectious colitis (*Clostridium difficile*)	Bile acids	(212)

4-EPS, 4-ethyl phenol sulfate; HBP, d-glycero-β-d-manno-heptose-1,7-biphosphate; SCFAs, short-chain fatty acids; TMAO, trimethylamine N-oxide.

At present, the colonization of infant gut microbiota has been widely recognized for its role in brain development and shaping the immune system. The communication between the normal gut microbiota and the host contributes to the metabolism and immunity in early life (214). It is worth mentioning that the lack of colonization of specific microbiota in early life may induce inflammatory or allergic diseases later, such as asthma and inflammatory bowel disease (IBD) (215). Interestingly, the maternal agricultural exposure during pregnancy can reduce the risk of asthma in the offspring (216). This may be mediated by the microbiota, because there is evidence that agricultural exposure in early life increases the diversity of the microbiota (217). Consistently, the use of antibiotics within the first 6 months of life increases the risk of asthma and allergies at 6 years of age (218). Besides, antibiotics administered to infant within 1 year of age are associated with the development of type 2 diabetes, type 1 diabetes, overweight, and eczema later, which is associated with infant microbiota (219–221). Shortly, these data support the connection between disturbances in the microbiota in early life and long-term health. However, the specific influencing factors and mechanisms need to be further explored.

### 5.4 Future Study Directions of Microbiota

#### 5.4.1 Characterizing Microbiome Composition

At present, it is possible to directly classify microbial populations without culturing due to the emergence of high-throughput DNA sequencing technology. Also, 16s RNA technology provides a reliable method for studying complex microbiota. Based on this, characterizing microbiome composition is the primary direction for future research on the role of microbiota in disease and health.

#### 5.4.2 Investigating the Function of Microbiota

Hypertranscriptome sequencing and macrogenomics laid the foundation for exploring the functions of the microbiota. The elucidation of the functions of the microbiota or specific microbes helps to regulate clearly the health or disease of the host.

#### 5.4.3 Causation or Correlation Between Microbiota and Host Disease

The treatment method based on micro-ecological failed to achieve the expected results, causing many conclusions in this field to be questioned. The article published by Nature explains that host variables can interfere with microbiome research, prompting researchers to fully consider host variables in the subsequent research process (222). Therefore, the causal relationship or correlation between the microbiota and the host diseases needs urgently to be studied in depth (223).

#### 5.4.4 Analysis of the Structure and Biological Activity of Microbial Metabolites or Components

The application of mass spectrometry and chromatography is of great significance for the exploration of microbial metabolites or components (224–226). Both targeted and untargeted proteomics and metabolomics are feasible for revealing the diversity of microbial metabolites or components. Exploring and identifying potential microbial metabolites or components provides opportunities for diagnosing and treating associated diseases.

#### 5.4.5 Isolation of Individual Strains

The isolation of individual strains or species in microbial communities has always been a challenge. Accurately capturing and analyzing the characteristics of individual microbial species is necessary for the implementation of research on microbial communities.

#### 5.4.6 Microbial Trace

The complex interaction between the microbiota and the host is a difficult point in the study. The trace of microbes provides “visual proof” for the ecological specificity of them. This method is expected to have a thorough understanding of the mechanism of action of the microbiota in the host.

## 6 Conclusions

The maternal microbiota are closely associated with the health of the fetus, and their disorder can lead to adverse pregnancy outcomes, including late abortion (LA), preterm rupture of membranes (PROM), hyperemesis gravidarum (HG), premature delivery (PTD), intrauterine growth restriction, and stillbirth. The maternal microbiota would be transmitted vertically to the newborn. The birth method, gestational age at birth, feeding method, and maternal factors all determine the colonization of the infant microbiota. The gut microbiota is the most important in the infant microbiota, and their cores are *Bifidobacteria*, the genus *Lactobacillus*, the genus *Clostridium*, the genus *Bacteroides*, the genus *Veillonella* and *Streptococcus*, and the genus *Collinsella* and *Akkermansia*. The microbiota in early life is essential for the brain development of offspring and the shaping of the immune system, which affects both infant health and long-term health. Therefore, from pregnancy to early life, reasonable intervention is important to regulate the maternal or offspring microbiota for offspring’s health.

## Author Contributions

YY and XC drafted the manuscript and assisted in reviewing literature. FW and YQY modified the manuscript. FC and CZ reviewed and edited the final manuscript. All authors contributed to the article and approved the submitted version.

## Funding

This study was supported by the National Natural Science Foundation of China (Grant Nos. 82003761 and 81873838) and Zhejiang Provincial Natural Science Foundation of China (Grant No. LY16H160025).

## Conflict of Interest

The authors declare that the research was conducted in the absence of any commercial or financial relationships that could be construed as a potential conflict of interest.

## Publisher’s Note

All claims expressed in this article are solely those of the authors and do not necessarily represent those of their affiliated organizations, or those of the publisher, the editors and the reviewers. Any product that may be evaluated in this article, or claim that may be made by its manufacturer, is not guaranteed or endorsed by the publisher.
